# Validation of the VisionArray® Chip Assay for HPV DNA Testing in Histology Specimens of Oropharyngeal Squamous Cell Carcinoma

**DOI:** 10.1007/s12105-024-01628-3

**Published:** 2024-03-27

**Authors:** Hani Ibrahim Channir, Simone Kloch Bendtsen, Linea Cecilie Melchior, Pia Rovsing Sandholm, Christine Mordhorst, Amanda-Louise Fenger Carlander, Christian von Buchwald, Katalin Kiss

**Affiliations:** 1 Department of Otorhinolaryngology, Head and Neck Surgery and Audiology, Copenhagen University Hospital-Rigshospitalet, Inge Lehmanns Vej 8, 2100 Copenhagen, Denmark; 2 Department of Pathology, Copenhagen University Hospital-Rigshospitalet, Copenhagen, Denmark

**Keywords:** Human papillomavirus, HPV DNA, Oropharyngeal cancer, Squamous cell carcinoma, VisionArray, HPV genotyping

## Abstract

**Background:**

The detection of human papillomavirus (HPV) has several implications in the diagnostic work-up and management of oropharyngeal squamous cell carcinoma (OPSCC). The choice of HPV detection assay and testing algorithms differ across institutions and vary in cost, detection targets, technical feasibility, and turnaround time. In this study, we aimed to validate the VisionArray® HPV Chip for formalin-fixed and paraffin-embedded (FFPE) samples of OPSCC using the previously applied standard pan-HPV DNA PCR assay as a reference.

**Methods:**

The validation cohort consisted of FFPE tissue samples from patients previously diagnosed with HPV DNA-positive OPSCC (n = 80), HPV DNA-negative OPSCC (n = 21), and a benign group of tumor samples consisting of Warthin’s tumors (n = 20) and branchial cleft cysts of the lateral neck (n = 14). All samples were tested with p16 immunohistochemistry, pan-HPV DNA PCR, and the VisionArray® HPV Chip.

**Results:**

The overall sensitivity and specificity of the VisionArray® HPV Chip assay were 100% [95% CI 95.5%; 100.0%] and 96.3% [95% CI 87.3%; 99.6%] and the positive predictive value and negative predictive value were 97.6% [95% CI 91.5%; 99.7%] and 100% [95% CI 93.2%; 100%], respectively.

**Conclusions:**

The VisionArray® HPV Chip assay can be recommended for high-risk HPV testing in FFPE tissue samples from OPSCC, providing both a fast and simultaneous genotyping for 41 clinically relevant HPV types.

## Introduction

The detection of human papillomavirus (HPV)-related oropharyngeal squamous cell carcinoma (OPSCC) is increasingly important in the routine clinical setting. Establishing the HPV status adds valuable information for staging and prognostication of patients with OPSCC. Additionally, in cytology and histology specimens from patients with cervical squamous cell carcinoma metastasis and unknown primary tumor, detection of oncogenic (or high-risk) HPV can further guide clinicians to the primary tumor origin (i.e., palatine and lingual tonsils) [1]. Many clinical trials are currently investigating new treatment modalities and de-escalation schemes for HPV-related OPSCC and may demand an upfront HPV status prior to inclusion [2]. Recent data from the multinational and multicenter EPIC study have shown that discordant HPV status in OPSCC (p16−/HPV + or p16 + /HPV−) have a significantly worse prognosis than patients with p16 + /HPV + OPSCC and therefore recommend specific HPV testing being performed in clinical trials along with p16 immunohistochemistry (IHC) [3]. HPV testing is most often performed by surrogate marker p16 IHC, as it is recommended by the College of American Pathologist (CAP) [4], and rarely in combination with high-risk (HR)-HPV testing using DNA PCR or E6/E7 mRNA in situ hybridization (ISH). mRNA ISH is considered the gold standard as it determines transcriptionally active HPV.

Current p16 and HPV testing guidelines in head and neck cancer are mainly based on the CAP evidence-based guidelines from 2018, which states that all new OPSCC patients should be tested with p16 IHC with a 70% nuclear and cytoplasmic positivity as cutoff [3]. During the past decade, our institution has continuously implemented and validated assays for PCR-based HPV testing that could be applied for both histology and cytology specimens [1, 5, 6]. Currently, we have a database with nearly 3000 OPSCC patients, which have been previously tested for p16 and HPV DNA including data on HPV genotyping [7, 8]. In the pursue of an in-house HPV-specific assay for routine clinical use in combination with p16 immunohistochemistry, we implemented a PCR-based DNA assay, VisionArray® HPV Chip, in 2017, which allows for simultaneous genotyping of 41 clinically relevant HPV types. However, it has not been validated for head and neck squamous cell carcinomas and therefore we aimed to validate the assay for formalin-fixed and paraffin-embedded (FFPE) samples of OPSCC using the previously applied standard pan-HPV DNA PCR as a reference.

## Materials and Methods

### Patients and Samples

This retrospective study retrieved archived FFPE samples from patients diagnosed with OPSCC (n = 101) between 2018 and 2019 (either HPV DNA-positive or negative) and a benign group of tumor samples consisting of Warthin’s tumors (adenolymphoma, n = 20) diagnosed between 2013 and 2014 and branchial cleft cysts of the lateral neck (n = 14) diagnosed between 2013 and 2015. Samples consisted of a biopsy or resection specimens and were reviewed by expert head and neck pathologists at the Department of Pathology, Copenhagen University Hospital—Rigshospitalet, Denmark. All patients with OPSCC were tested with p16 IHC in which strong and uniform p16 staining (both cytoplasmic and nuclear) in > 70% of tumor cells was considered positive.

### Extraction of DNA

DNA was extracted and purified from one to four 10-μm slices of FFPE samples using either an automated QIAcube and Qiagen’s GeneRead DNA FFPE Kit (#180134, Qiagen, Hilden, Germany) according to the manufacturer’s recommendations (OPSCC samples) or by manual extraction (all other samples and any secondary DNA extractions). For the manual extraction, 200 µL Tris–EDTA buffer solution was added to the FFPE slices before melting the paraffin at 95 °C for 10 min while gently vortexing. The samples were cooled to 56 °C, making the paraffin gather as a solid layer on top of the sample, and 20 µL Proteinase K (Qiagen) was added through a small hole in this layer. Finally, the samples were incubated over night at 56 °C while gently vortexing. The DNA concentration was measured using either DeNovix DS-11 (DeNovix, Wilmington, DE, USA) or NanoDrop (ThermoFischer, Waltham, MA, USA).

### Pan-HPV DNA PCR of OPSCC Samples

HPV status of OPSCC samples was assessed by initial pan-HPV testing by PCR using the general primers GP5+/6+ as described earlier [9]. DNA quality was confirmed with a GAPDH control. PCR products were visualized on a pre-cast 2% agarose E-gel (Invitrogen, Waltham, MA, USA) using the Gel Doc EZ system (Bio-Rad, Hercules, CA, USA). The image was assessed with the Molecular Imager and Image Lab software (both from Bio-Rad) according to the manufacturer’s instructions. The expected amplicon sizes were approximately 150 base pairs for GP5+/6+ and 200 base pairs for GAPDH. Samples positive for GP5+/6+ and GAPDH were deemed HPV-positive, and samples negative for GP5+/6+ but positive for GAPDH were deemed HPV-negative. The turnaround time for this assay was a mean of four calendar days [1].

### VisionArray® HPV Chip Assay

All samples were analyzed for HPV status and genotype using the VisionArray® HPV Chip 1.0 system (#VA-0001, ZytoVision, Bremerhaven, Germany). For the OPSCC specimens, material from the same DNA extraction as for the initial pan-HPV DNA PCR assay was used. The VisionArray® HPV Chip detects DNA from 41 clinically relevant HPV genotypes classified as 12 high risk (HPV16, 18, 31, 33, 35, 39, 45, 51, 52, 56, 58, and 59), 12 probably high risk (HPV26, 34, 53, 66, 67, 68a, 68b, 69, 70, 73, 82IS39, and 82MM4), and 17 low risk (HPV6, 11, 40, 42, 43, 44, 54, 55, 57, 61, 62, 72, 81CP8304, 83MM7, 84MM8, 90, and 91) according to the manufacturer. The assay was performed according to the manufacturer’s recommendations using the VisionArray HPV PreCise Master Mix (ZytoVision #ES-0007) followed by the detection kit (ZytoVision, #VK-0003). Chip scans were analyzed using the VisionArray MultiScan E4302 software with a threshold of 25. The turnaround time for this assay was up to 24 h.

### Statistics

Statistical analyses calculating the sensitivity, specificity, positive and negative predictive values were performed using IBM SPSS Statistics for Windows, Version 28.0. Armonk, NY: IBM Corp.

## Results

**Fig. 1:**
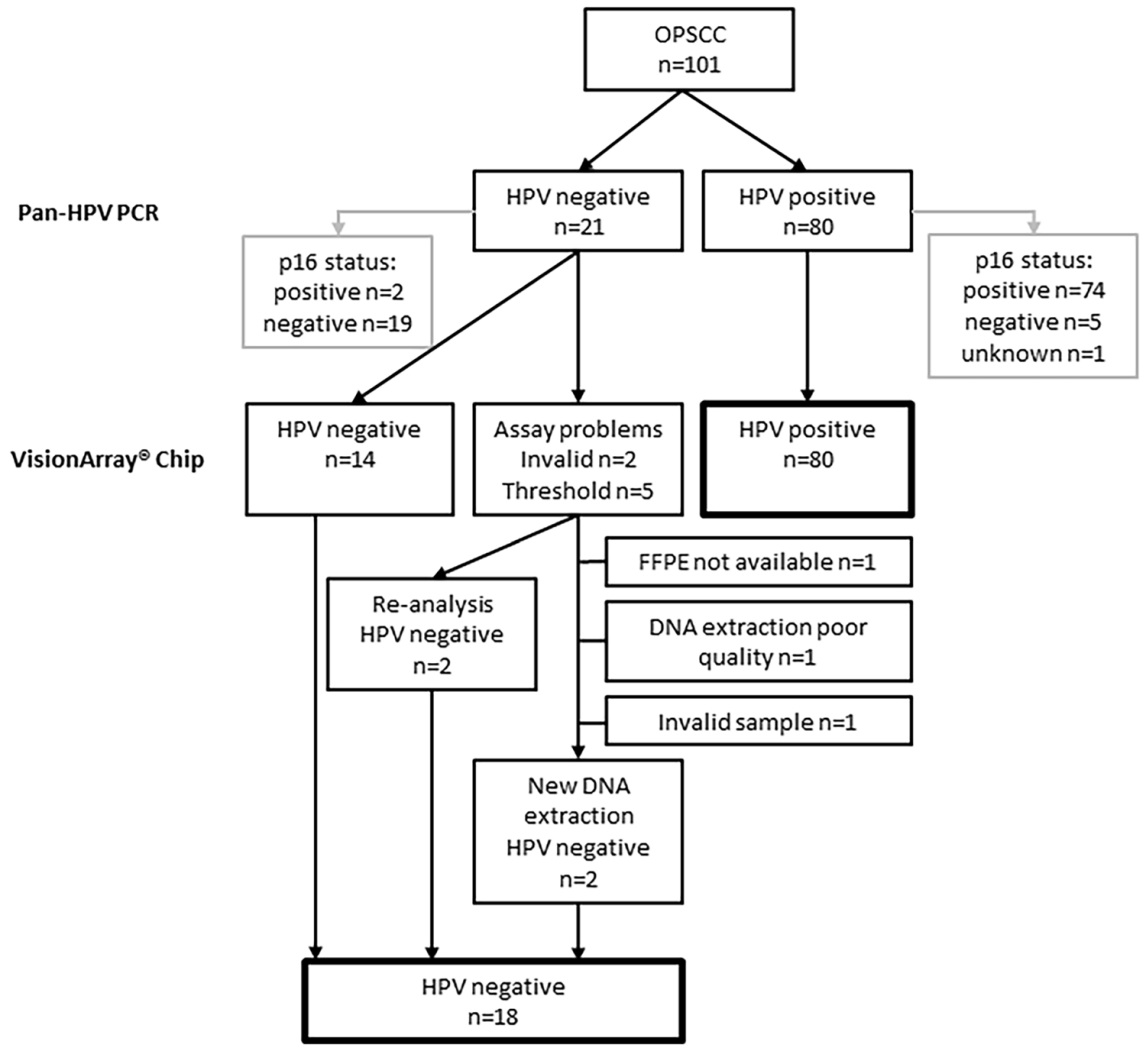
Flowchart illustrating all samples of oropharyngeal squamous cell carcinoma analyzed by p16 immunohistochemistry, pan-HPV DNA PCR, and the VisionArray® HPV Chip

**Fig. 2:**
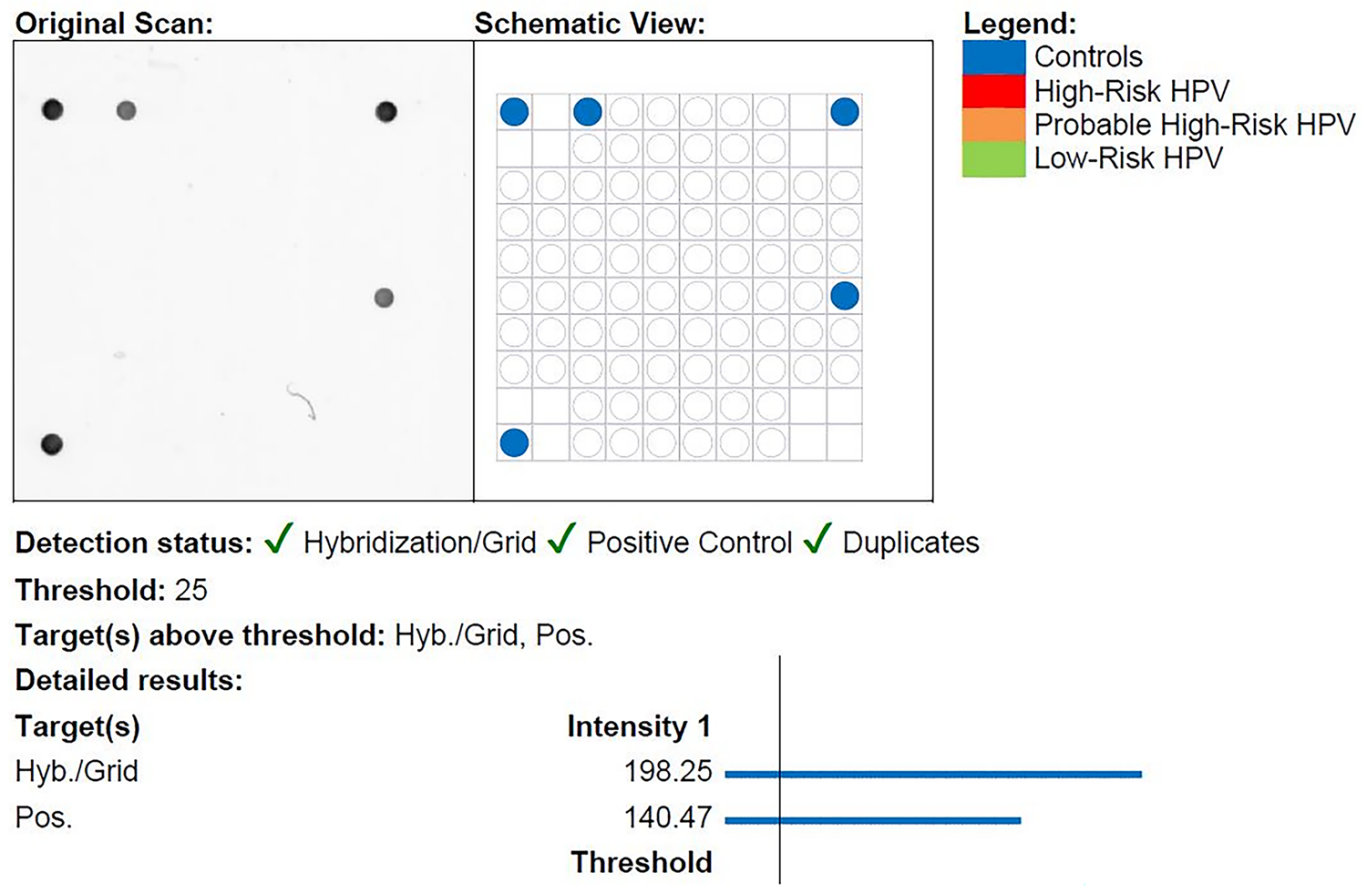
Showing an HPV-negative sample analyzed by the VisionArray® HPV Chip including both the original chip scan and the schematic view. Blue dots in the schematic view illustrates the positive controls meeting threshold requirements

**Fig. 3:**
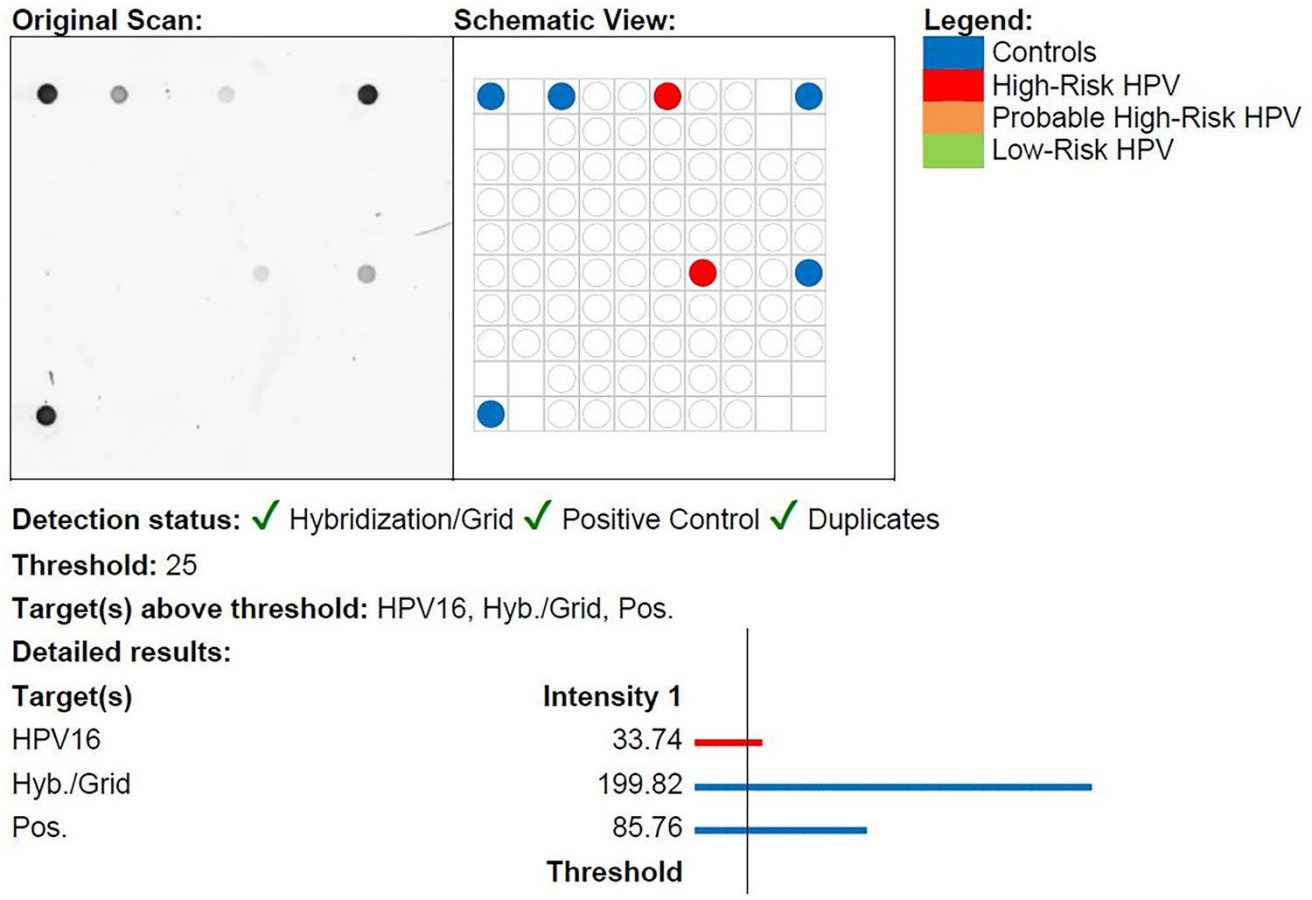
Showing an HPV-positive sample analyzed by the VisionArray® HPV Chip including both the original chip scan and the schematic view. The latter illustrates the positive controls (blue dots) and HPV16 positivity (red dots) meeting threshold requirements

We included samples from 101 patients with OPSCC; the average age was 61 years (range: 40–84 years) with the majority being male (72%). Eighty samples were HPV-positive using the pan-HPV PCR of which 74 samples were corresponding p16-positive, five were p16-negative, and one had unknown p16 status due to lack of remaining tumor material. All 80 samples were correspondingly HPV-positive using the VisionArray® HPV Chip as illustrated in the flowchart in Fig. 1. The predominantly tested genotype was HPV-type 16 (n = 71, 89%), followed by HPV33 (n = 5), HPV18 (n = 1), HPV35 (n = 1), HPV59 (n = 1), and HPV67 (n = 1). An example of an HPV-type 16-positive and HPV-negative chip scan using the VisionArray® HPV Chip is illustrated in Figs. 2 and 3.

Twenty-one patients were HPV DNA-negative using the pan-HPV DNA PCR of which corresponding p16 was negative in 19 and positive in two samples. With the VisionArray® HPV Chip, 18 samples were tested HPV-negative (including the mentioned p16-negative/HPV DNA-positive samples), four of these after repeated analysis since the first analysis did not meet the threshold requirements. Three samples were excluded due to insufficient DNA quality or lack of FFPE material for DNA extraction.

A total of 20 Warthin’s tumors of the salivary glands and 14 branchial cleft cysts of the neck were included, all of which tested HPV DNA-negative with both methods and were p16-negative.

### Sensitivity and Specificity

The overall sensitivity and specificity of the VisionArray® HPV Chip assay were 100% [95% CI 95.5%; 100.0%] and 96.3% [95% CI 87.3%; 99.6%] and the positive predictive value and negative predictive value were 97.6% [95% CI 91.5%; 99.7%] and 100% [95% CI 93.2%; 100%], respectively.

## Discussion

The VisionArray® HPV Chip was successfully implemented in our laboratory with good analytical capabilities illustrated by the high sensitivity and specificity in comparison to our previously used standard reference assay, pan-HPV DNA PCR which had a sensitivity of 86.7% and specificity of 92% as shown previously [1]. Another advantage of the VisionArray® HPV Chip assay is that it provides simultaneous HPV genotyping as a same-step procedure detecting 41 clinically relevant genotypes classified as low risk, probably high risk, and high risk. In the current validation cohort, the most frequently detected HPV genotype was HPV16 which reflects the demographic patient group from Eastern Denmark [7, 8]. Prior to this study, we had to acquire the genotype as a secondary analysis using next-generation sequencing which was more time consuming and expensive.

To our knowledge, HPV genotyping does not have clinical implications in terms of treatment or prognostication but it may provide important information for research purposes in epidemiologic studies and in the etiopathogenesis of OPSCC. In future, it may add value in relation to liquid biopsies with cell-free HPV DNA in the follow-up surveillance after treatment [10]. A recent study has investigated the role of high-risk HPV genotypes on survival in patients with oropharyngeal cancer, and found no differences when comparing HPV16 with non-HPV16 genotypes [11]. The subgroup analysis indicated that the group of patients with genotypes HPV33 and HPV35 has a significantly better 5-year overall survival than other non-HPV16 genotypes.

Advanced HPV testing including genotyping can be adopted by the pathologist in other head and neck entities. It has an important role in the diagnostics of sinonasal malignancies, in particular the HPV-related Multiphenotypic Sinonasal Carcinoma, which requires the presence of certain HPV genotypes [12]. The 5th edition of the world health organization classification of head and neck tumors recommends that high-risk HPV must be demonstrated by in situ hybridization or PCR-based techniques, specifically to include type 33. p16 alone is not sufficiently specific to make the diagnosis [13].

HPV genotyping is often requested by otolaryngologists after excision of respiratory papillomas where approximately 30% of head and neck papillomas are related to HPV [14]. Establishing low-risk HPV genotypes such as HPV6 and HPV11 plays an important role during the patient consultation.

The VisionArray® HPV Chip assay has proven to be highly suitable in clinical practice, as the HPV testing can be performed within 24 h compared with the more time-consuming pan-HPV DNA PCR where the turnaround time is up to four days. This is especially useful if applied on cytology specimens, and if HPV-positive, it facilitates the diagnostic work-up and treatment for patients who present with a metastatic squamous cell carcinoma of the neck and unknown primary. HPV DNA testing is feasible on previously stained cytology smears as previously shown [1, 5]. The use of p16 staining on cytology specimens is not recommended as there is no validated cutoff value and it requires the preparation of a cell block, with a risk of not having viable tumor cells [15].

This study was strengthened by the large validation cohort including both HPV-positive and negative OPSCC, and benign tumors previously tested for HPV DNA. Among the HPV-negative patient samples, we experienced a few samples that had either poor DNA quality or insufficient DNA concentration for HPV testing which could be explained by several factors. Firstly, we utilized 4- to 5-year-old archived FFPE material where the DNA quality can be varying, and secondly, some samples consisted of very small biopsies with none or limited tumor tissue left after being used for p16 staining and the reference pan-HPV DNA PCR assay. As part of every implementation process, we must highlight the importance of the pre-analytical steps in the laboratory to avoid contamination and cross-contamination between patient samples that could lead to inaccurate results.

The HPV testing algorithms and choice of assay vary among laboratories across the world and are dependent on the resources, capacity, and staff at the different institutions. A full review of all HPV testing assays is beyond the scope of this paper; nonetheless, the most commonly used targets are HPV DNA, HPV RNA, viral oncoproteins, cellular proteins, and HPV-specific serum antibodies [16, 17]. In the CAP guidelines, p16 IHC is recommended for biopsy/resection specimens and is the most widely used surrogate marker for HPV [4]. The guidelines further state that additional HPV-specific testing may be done based on the decision of the pathologist and/or treating clinician, or in the context of a clinical trial. p16 IHC is suitable as a stand-alone assay in most clinical settings for histologic specimens deriving from oropharyngeal tumors as it provides acceptable sensitivity and specificity, is much more cost-effective than molecular tests, has a short turnaround time, and is easy to analyze. However, the additional use of specific HPV DNA testing should be strongly considered for patients that are p16-positive and potential candidates for clinical trials that offer either de-escalation or intensification of treatment. This is based on a recently published multinational study which investigated discordant p16/HPV oropharyngeal cancers and its prognostic implications [3]. The authors argue that an exception could be made in some geographical regions which is associated with a high p16/HPV concordance (i.e., North America). Interestingly, the study showed that if p16 IHC alone is used to determine HPV status, 8.1% of p16-positive patients worldwide and up to almost 26% in regions of low HPV-attributable fractions such as southern Europe would be incorrectly classified as having HPV-related tumors.

Therefore, at our institution, HR-HPV testing is currently being applied on either a resection specimen of a metastasis or on the primary tumor (biopsy/resection) along with p16 IHC as the majority of our patients participate in clinical trials. In the few discordant p16-positive and HPV DNA-negative cases, we request HPV genotyping at a different laboratory as a quality assessment, to ensure that we do not fail to detect a genotype that is not detected by the VisionArray® HPV Chip assay which has not been an issue to date.

The recommended gold standard method for HR-HPV testing is the mRNA ISH which detects transcriptionally active HPV E6/E7 oncogenes. This assay has previously been validated against p16 and HPV DNA by several institutions who agree on its excellent analytical capabilities, technical feasibility, and fast turnaround [6, 18–21]. Furthermore, it provides direct visualization of the HPV-positive staining which further strengthens the sensitivity and specificity. However, the assay is limited by its use on research platforms only; it is less cost-effective than p16 IHC and PCR-based assays and currently only allows for the detection of mRNA of up to 18 HR-HPV genotypes in a single cocktail probe. Based on our validation cohort, it would have failed to detect HPV-type 67 in a single patient which is classified as a “probably high risk” HPV type using the VisionArray® HPV Chip. Another limitation is that the ISH method requires a morphologically well-preserved specimen where the cell nuclei are well visualized. On the contrary, the PCR-based analysis works well on crushed tumor cells like the defrosted frozen section tissue specimen or laser coagulated tissue specimens. In the future, it is necessary to clarify when to perform HPV mRNA ISH in routine clinical use in relation to the existing assays.

In conclusion, we found that the VisionArray® HPV Chip assay can be recommended for HR-HPV testing in FFPE tissue samples from OPSCC providing both a fast and simultaneous genotyping for 41 clinically relevant HPV types.

## Data Availability

All data analyzed during this study are included in this article. Further enquiries can be directed to the corresponding author.

## Abbreviations

OPSCC: Oropharyngeal squamous cell carcinoma
HPV: Human papillomavirus
FFPE: Formalin-fixed and paraffin-embedded
